# Role of indigenous iron in improving sludge dewaterability through peroxidation

**DOI:** 10.1038/srep07516

**Published:** 2015-01-06

**Authors:** Xu Zhou, Guangming Jiang, Qilin Wang, Zhiguo Yuan

**Affiliations:** 1 Advanced Water Management Centre, The University of Queensland, St. Lucia, Queensland 4072, Australia

## Abstract

Improvement of sludge dewaterability is important for reducing the total costs for the treatment and disposal of sludge in wastewater treatment plants. In this study, we investigate the use of hydrogen peroxide as an oxidizing reagent for the conditioning of waste activated sludge. Significant improvement to sludge dewaterability was attained after the addition of hydrogen peroxide at 30 mg/g TS and 28 mg/g TS under acidic conditions (pH = 3.0), with the highest reduction of capillary suction time being 68% and 56%, respectively, for sludge containing an iron concentration of 56 mg Fe/g TS and 25 mg Fe/g TS, respectively. The observations were due to Fenton reactions between the iron contained in sludge (indigenous iron) and hydrogen peroxide. For the sludge with an insufficient level of indigenous iron, the addition of ferrous chloride was found to be able to improve the sludge dewaterability. The results firstly indicated that indigenous iron can be utilized similarly as the externally supplied iron salt to improve sludge dewaterability through catalyzing the Fenton reactions.

As an effective method, the activated sludge process has been applied to wastewater treatment for over a century. One well-known problem for the process is the high cost associated with the treatment and disposal of waste activated sludge (WAS) produced during the treatment process. Sludge treatment and disposal may account for up to 60% of the total operational costs in a wastewater treatment plant (WWTP)^1^.

The sludge treatment process usually consists of five steps: thickening, stabilization, conditioning, dewatering, and disposal/reuse. Among them, the dewatering process reduces the sludge volume by separating water from sludge solids. The water in WAS could be classified as free water and bound water^2^. The former combines with the sludge loosely and could be easily removed with the mechanical dewatering methods. In contrast, the bound water is hard to be removed mechanically because it combines with the sludge structure by capillary forces or chemical bonds. To increase the WAS dewaterability, sludge conditioning is used widely as a pretreatment. Many conditioning approaches, involving physical, chemical or enzymatic treatment of sludge, have been developed for improving the WAS dewaterability.

Heat treatment (thermal hydrolysis), as a physical method, could denature the proteins in extracellular polymeric substances (EPS) and disintegrate the cells^3^,^4^. Also, the hydrolysis of extracellular and intracellular compounds leads to the decomposition of the sludge structure and then the release of the bound water. Similarly, the freezing-thawing method involves reducing the bound-water content of the sludge, and thus restructuring the sludge flocs into a more compacted and denser form. Physical treatment also includes various mechanical methods such as ultrasonic treatment and pressure homogenization^5^.

Chemical methods, through the addition of chemicals such as flocculants, acids or alkali, increase the flocculation ability of the sludge and facilitate the release of bound water^6^,^7^. Similar to the physical disintegration methods, chemical treatment help the release of water held by flocs and cells which cannot be removed by the conventional dewatering processes^8^. Chemical methods sometimes are also combined with physical treatment. The frequently used acid thermal hydrolysis method is such an example. However, some chemical conditioning methods cause secondary environment pollution. For example, polymer flocculation agents are hard to be degraded in natural environment, thus posing potential environmental risks^9^.

Another type of chemicals used for increasing dewaterability is the strong oxidizing agents, such as Fenton reagent, Fenton-like reagents and ozone. The oxidization treatment of WAS may partially dissolve sludge flocs, in addition to decreasing the surface charges of sludge particles^10^,^11^,^12^. Hydrogen peroxide is already widely applied as the oxidizing agent. Its advantage includes high oxidation efficiency, low cost and no residuals after the reactions^13^.

The classical Fenton reaction consists of a series of reactions occurring between ferrous Fe(II) ions and hydrogen peroxide in liquid under acidic condition (Eq.1–10). The reactions produce hydroxyl radicals (Eq.1), which oxidize the target compounds. Fenton-like reactions could also take place between ferric Fe(III) ions and hydrogen peroxide. On the other hand, some types of chelator could form complexes with ferrous and ferric ions in liquid by its carboxylate functional groups, thus facilitating the Fenton reaction^14^.


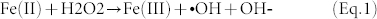

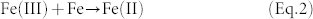

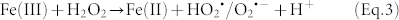

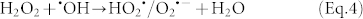

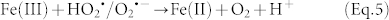

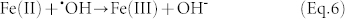

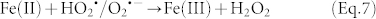

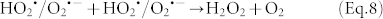

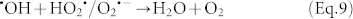

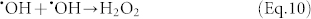


The use of Fenton reaction to improve sludge dewaterability through the simultaneous addition of hydrogen peroxide and iron salts has been studied by several researchers previously^11^,^12^,^15^.With the simultaneous addition of ferrous salt, Kim et al., showed that sludge peroxidation enhanced the disintegration of sludge particles, and hence improved the sludge solubility (SCOD/TCOD) and settleability, in addition to decreasing sludge viscosity^16^. Neyens et al., reported sludge peroxidation treatment reduced the amount of sludge by 50% and improves the dry solids content in sludge cake by 100%^13^.

However, various levels of iron may be present in the WAS, depending on upstream wastewater sources or treatment processes. Industrial wastewater containing high concentration of iron can be discharged into domestic sewers. Iron salts may also be added for the control of hydrogen sulfide in sewers or for phosphorus removal in the wastewater treatment plants^17^. If the indigenous iron, originated from upstream sewer or wastewater treatment plant, is available for the Fenton reaction, the amount of iron salts to be added for sludge conditioning could be reduced or even become unnecessary. However, such kind of phenomenon has never been studies before.

In this study, we firstly investigate if the indigenous iron in activated sludge is available for the Fenton reaction following the addition of hydrogen peroxide, which would consequently improve the dewaterability of WAS. Two sludge sources with different concentrations of indigenous iron were used in the experimental study. Capillary suction time (CST), which stands for the time needed for completing the sludge filtration process, is measured as the indicator of sludge dewaterability. Other parameters like iron concentrations in sludge and filtrate were also measured to shed light on the mechanisms involved.

## Results

### Effectiveness of peroxidation on improving dewaterability

**Figure 1:**
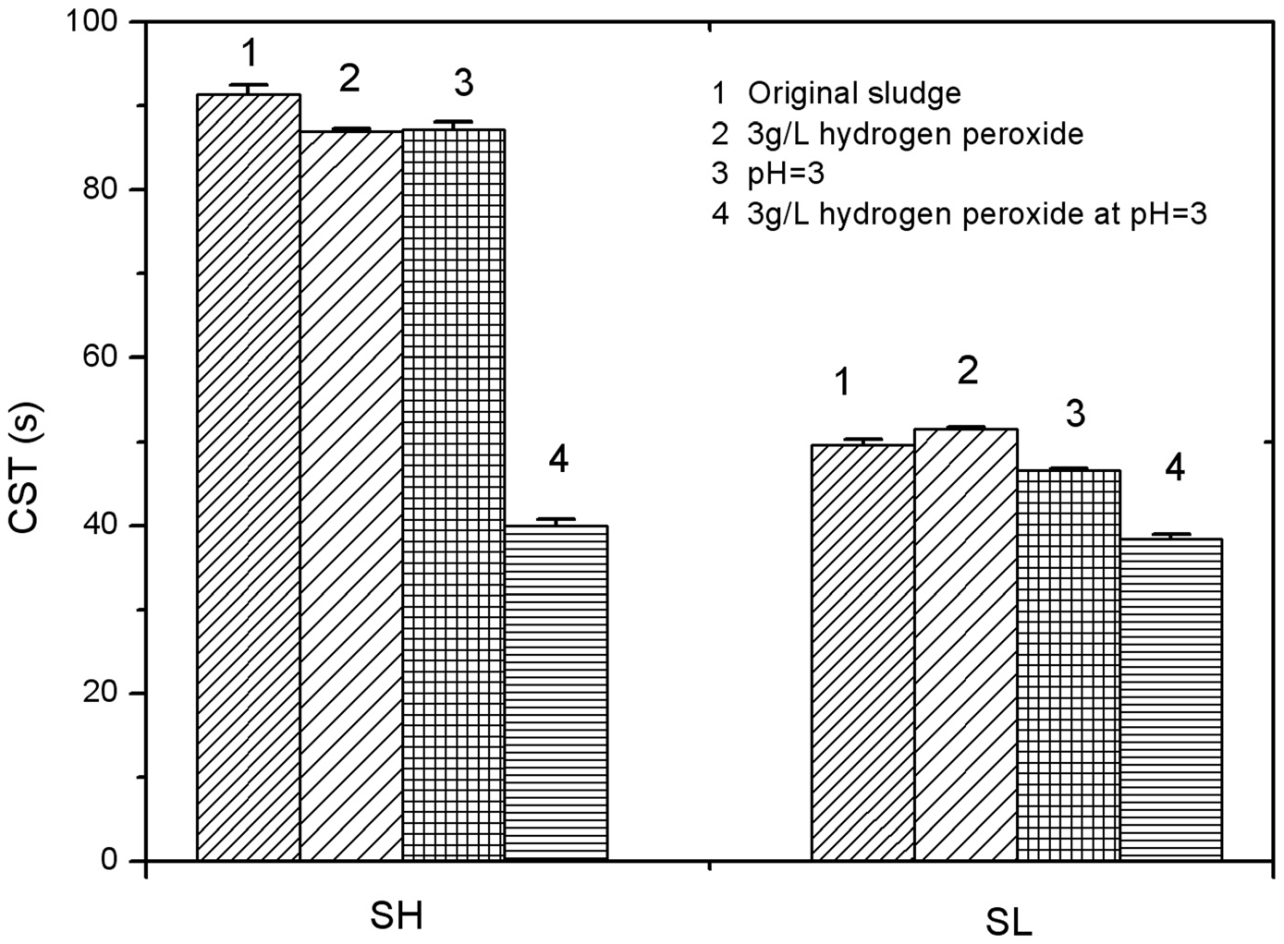
Performance of peroxidation on enhancing dewaterability of waste activated sludge SH and SL.

Figure 1 shows the change of sludge dewaterability before and after being treated with hydrogen peroxide (3 g/L), acidic pH (3.0) or both (Test Group I).

According to Fig. 1 and Table 1, it was found that CST of SH was longer than SL. At the same time, the TS in SH was only slightly lower than SL. Many factors could affect the sludge dewaterability, including the flocculability and structure of sludge flocs. The sludge dewaterability could be deteriorated if the sludge contained higher amount of fine particles, even with lower TS content. The two kinds of sludge were collected from two different WWTPs, whose operation conditions may also contribute to the different initial CST values.

For both sludge SH and SL, with the adjustment of pH to 3.0 from their original levels, i.e. 6.8 and 6.1 for SH and SL respectively, the CST only decreased slightly by 5% and 6%. Similarly, the CST only decreased slightly by 5% and 4% for SH and SL, respectively, after adding 3 g/L of hydrogen peroxide without pH adjustment.

However, when hydrogen peroxide was added in the acidified sludge (pH = 3), CST values decreased significantly for both sludges. Especially, CST for sludge SH decreased from above 90 sec to 40 sec, implying a great improvement of sludge dewaterability. For sludge SL, its dewaterability was reduced from 50 s to 38 s, a 23% reduction compared to the 56% reduction achieved for sludge SH. These results indicate that the level of dewaterability improvement by sludge peroxidation depends on low pH and also on sludge properties. Sludge SH achieved a higher improvement of dewaterability than sludge SL, which is likely related to the different concentration of indigenous iron concentration. This is because one major mechanism of sludge peroxidation is the Fenton reaction, which is generally catalyzed by iron. Sludge SH has 525 mg/L of iron, which is almost 4 times higher than the iron concentration (107 mg/L) in sludge SL (Table 1). Due to the abundance of iron in SH, it has likely experienced more effective treatment due to higher Fenton activity, therefore achieving higher reduction of CST. The solid contents in sludge before and after peroxidation treatment were also measured, but the values were almost unchanged (P > 0.05). This might be due to the fact that the oxidization process changed the floc structure rather than eliminating the flocs itself. The water content in sludge floc during the peroxidation process was also unchanged. However, the water could be separated more easily from the flocs, which was also shown by the CST values.

### Effects of iron concentration

**Figure 2:**
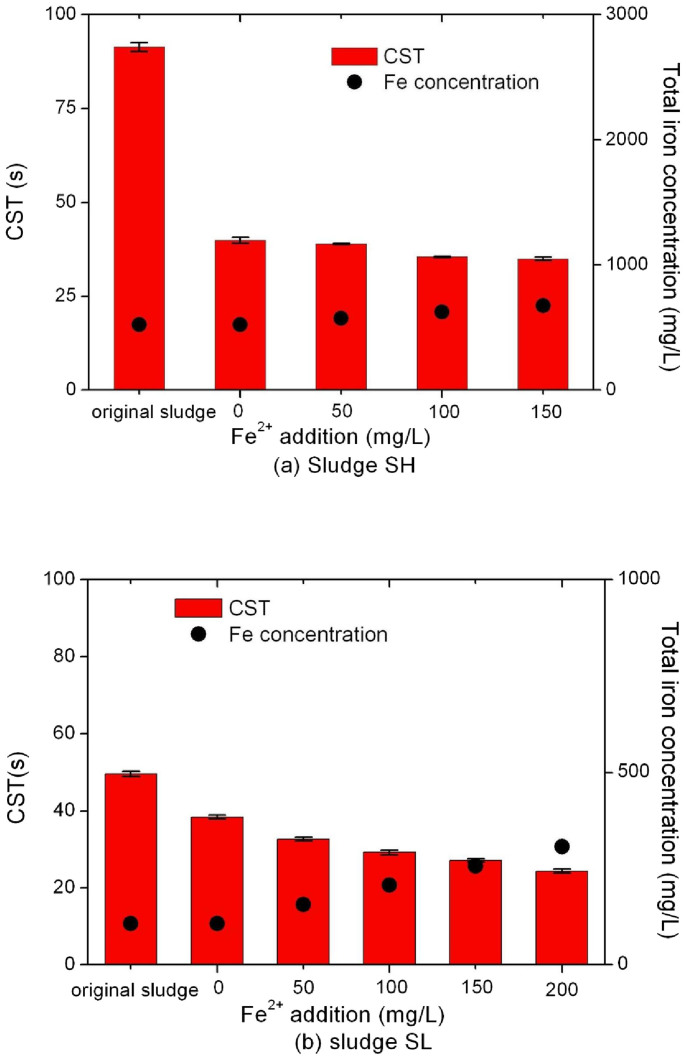
Effect of ferrous chloride addition on the dewaterability of sludge SH (a) and sludge SL (b). The hydrogen peroxide concentration used was 3000 mg/L and pH was 3.0 in all tests.

The results of Test Group 1 confirmed the effectiveness of peroxidation on enhancing sludge dewaterability, with higher improvement for sludge SH with a high indigenous iron concentration. Tests 8–12 were thus designed to investigate the effects of iron addition on the efficacy of peroxidation. Figure 2 shows the improvement of sludge dewaterability after peroxidation treatment with various levels of iron addition. The hydrogen peroxide concentration was at 3000 mg/L, which was shown to be effective in improving sludge dewaterability (Test Group I). For sludge SH, CST was marginally improved from 40 s to 35 s while the total concentration of iron in sludge was increased from 525 mg/L (concentration of indigenous iron) to 625 mg/L (56 mg Fe/g TS). However, addition of a higher amount of iron to 675 mg/L did not further reduce the CST value (*P* = 0.0514). In contrast, the dewaterability of sludge SL gradually increased with the increase of addition of iron up to 200 mg/L (Fig. 2b). The CST was reduced from 38 s to 24 s when the total iron concentration in sludge was increased from 107 to 307 mg/L (25 mg Fe/g TS). A significant decrease in CST was observed after each increased addition of ferrous chloride (*P* < 0.001 in all cases). The results clearly show the important role of iron in the improvement of sludge dewaterability.

**Figure 3:**
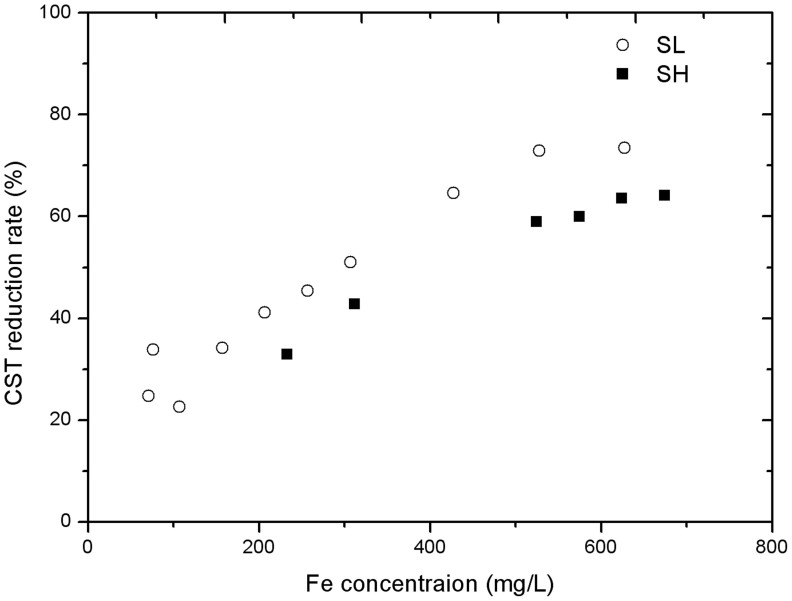
The dependency of CST reduction on the concentration of iron observed in Test Group II. In all tests, the concentration of hydrogen peroxide was 3000 mg/L, and pH was 3.0.

To further verify the role of indigenous iron in dewaterability improvement, Tests 4–7 were designed, in which part of the iron in the original sludge was removed through leaching. Figure 3 summarizes the improvement of sludge dewaterability (CST reduction percentage) obtained in these tests, based upon the total amount of iron available. For comparison, the CST results reported in Figure 3 are also plotted in Figure 3. It is evident that in all tests, peroxidation improved the sludge dewaterability. However, the effectiveness decreased with the decrease in iron concentration caused by iron removal through leaching. Overall, a clear dependency of CST reduction on the iron concentration was evident for both kinds of sludge.

The experimental results (Fig. 3) also showed that the highest CST reduction was achieved when the total sludge iron was higher than ~500 mg/L. Before reaching the maximum, the CST reduction percentage almost increased linearly with the increase of the total sludge iron concentration. This trend is the same for both SH and SL. After the iron leaching pretreatment, the concentration of iron in sludge SH was decreased to 312 mg/L and 233 mg/L respectively, while that in sludge SL was decreased to 76 mg/L and 71 mg/L. It is clear that these pretreated sludge showed lower dewaterability improvement after peroxidation, in comparison to the original sludge. Therefore, both the indigenous sludge iron and added iron contributed to the reduction of CST.

The results leads to the conclusion that both indigenous iron (at acidic pH) and additional iron could react with hydrogen peroxide to improve sludge dewaterability most likely through Fenton reactions. Further addition of ferrous salt can improve the effectiveness of peroxidation only when the indigenous iron concentration is low, such as in the case of sludge SL. Once the indigenous iron reached a high level (>500 mg/L), further addition of ferrous salt only improve marginally the dewaterability.

### Effects of hydrogen peroxide concentration

**Figure 4:**
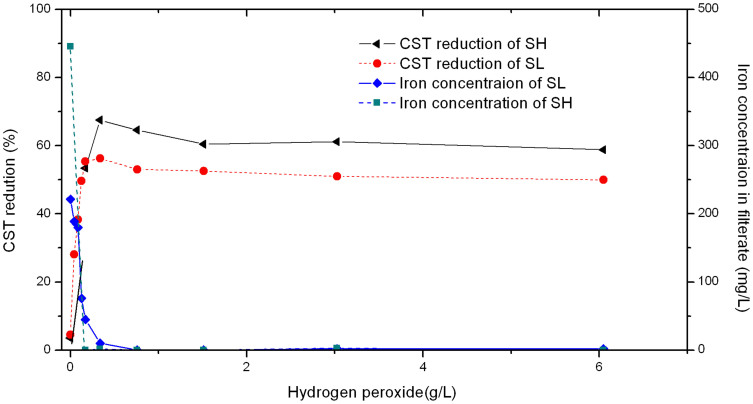
Effects of hydrogen peroxide concentration on sludge dewaterability after peroxidation treatment with fixed addition of ferrous salt at 100 and 200 mg/L for sludge SH and SL, respectively. Iron concentrations in the treated SL and SH filtrate are also presented.

Results from Test Group II showed that a sufficient amount of iron, either as indigenous iron or through external addition, is essential for the improvement of dewaterability. Test group III further investigated the effects of hydrogen peroxide concentration when a sufficient amount of iron was present in the sludge. Figure 4 shows the reduction of CST for sludge treated with fixed iron concentrations (Tests 13–27, for Sludge SH and SL, respectively) and various hydrogen peroxide concentrations.

When the concentration of hydrogen peroxide was around 340 mg/L, the highest reduction of CST was achieved for both sludge SH and SL. Sludge SH achieved 68% reduction of CST at 340 mg/L of hydrogen peroxide. For sludge SL, when the concentration of hydrogen peroxide increased from 40 to 340 mg/L, the CST reduction percentage also increased from 28% to 56%. For both sludges, the addition of more hydrogen peroxide did not achieve further improvement to the sludge dewaterability.

The concentration of iron in filtrate of the treated sludge was also measured. As shown in Fig. 4, the concentration of iron in filtrate decreased while the hydrogen peroxide concentration increased from 0 mg/L to 760 mg/L in Sludge SL. The total iron in filtrate remained around 0 mg/l while the hydrogen peroxide was higher than 1000 mg/L. The phenomenon may be due to the oxidization of Fe^2+^ to Fe^3+^, which would have formed ferric hydroxide precipitate.

## Discussion

The results demonstrated that acidic pH alone, or hydrogen peroxide alone could achieve very limited improvement to sludge dewaterability. The addition of hydrogen peroxide under acidic conditions significantly improved the sludge dewaterability, with the extent of improvement strongly dependent on the iron concentration. This is due to the initiation of Fenton process was caused by iron. The Fenton process produced hydroxyl radicals that partly oxidized sludge flocs (Eq.1–10). For sludge with a low concentration indigenous iron (e.g. <50 mgFe/gTS), an additional amount of iron salt is needed to improve the dewaterability through peroxidation treatment. These findings support that the improvement of sludge dewaterability could be achieved only when both Fe and hydrogen peroxide co-exist at sufficient concentrations under acidic conditions. This coincides with the conditions of Fenton reaction, which may indeed occur between the hydrogen peroxide and iron whether it is indigenous or added externally. Similar to other oxidization processes, Fenton oxidization could disrupt the flocs and cells, thus free the interstitial water from sludge structure.

Iron likely exists in most WAS. Iron could have been introduced in the treatment process, for example for phosphorus removal or as a flocculent^18^, or contained in wastewater. Iron in sludge plays an important role in maintaining the floc structure by combining with proteins^19^. This work identified an opportunity to reuse the iron in sludge as the catalyst of Fenton reaction for improving sludge dewaterability. Furthermore, the operation of the process is practically feasible. It only needs to measure the concentration of indigenous iron in sludge occasionally, and no further procedures are needed. The iron concentration of the WAS from a WWTP is usually stable (depending the iron level in wastewater), thus the operating intensity would not be increased.

After the conditioning, the acidic filtrate of the conditioned WAS also needs to be neutralised. A lot of methods have been developed to neutralize the acidic wastewater before discharge. The traditional methods were mainly based on limestone addition. More recently, new methods were also conducted including the application of sorbent materials and coagulation–flocculation process^20^,^21^.

According to the results, the optimum ferrous addition amount was 100 mg/L (8.9 mgFe/gTS) and 200 mg/L (16.3 mgFe/gTS) for sludge SH and SL, with hydrogen peroxide at 340 mg/L (30 mg/gTS and 28 mg/gTS) for both kinds of sludge. Thus an estimation of chemical costs could be carried out, the total costs for sludge conditioning was $27.9 and $37.3/tone TS. (The prices of FeCl2, 50% hydrogen peroxide and 98% sulfuric acid are $600, $200 and $100/tonne, respectively).

The exact economic outcome would depend on how the solid content of the dewatered sludge would increase after conditioning. With a common solid content of 15% for dewatered sludge and the sludge transport and disposal cost of $55/wet tone in Australia, the costs for sludge conditioning would be balanced if the solid contents of the dewatered SL and SH after conditioning increase from 15% to 16.1% and 16.7%, respectively. Thus the sludge with higher indigenous iron could be easier to save the expense of treatment.

## Methods

### Sludge sources and chemicals

Two full-scale activated sludges, namely Sludge I and Sludge II, were used in this study. Sludge I was recycled activated sludge collected from the Elanora WWTP (Gold Coast, Australia), which removes phosphorus chemically with the addition of ferric chloride. Sludge II was the WAS collected from the Luggage Point WWTP (Brisbane, Australia), which does not involve the addition of iron salts in its processes. Before being used in the tests, the sludge was settled by gravity for 48 h to increase the concentration. The main characteristics of both types of sludge are listed in Table 1. Based on their iron content, Sludge I and sludge II are denoted as SH (sludge with high indigenous iron) and SL (sludge with low indigenous iron), respectively.

Analytical grade FeCl_2_·4H_2_O (Sigma-Aldrich) was dissolved in milliQ water for preparing the Fe^2+^ stock solution (20 g Fe^2+^/L). The stock concentration of H_2_O_2_ (Chem-Supply) was 30%. Sulfuric acid (Sigma-Aldrich, 99.999%), after being diluted to 25%, was used to adjust the pH of sludge.

### Batch tests

Three groups of batch tests were carried out to evaluate the preliminary effects of hydrogen peroxide treatment (Group I), effects of sludge iron concentration (Group II), and effects of hydrogen peroxide concentration (Group III), respectively, on sludge dewaterability. The design of these tests is summarized in Table 2. All the batch tests were done in duplicate.

Test Group I consists of preliminary tests to evaluate the possible improvement to sludge dewaterability by the addition of hydrogen peroxide to acidified sludge (pH = 3) and sludge without pH adjustment. The effect of sludge acidification (pH = 3.0) without hydrogen peroxide addition was also tested. Iron salt was not added in any of these tests. The dewaterability of sludge after treatment was measured after tests with methods to be described in 2.3.

Based upon the effectiveness of H_2_O_2_ on sludge dewaterability observed in Test Group I, Test Group II was designed to evaluate the availability of iron ions on the treatment performance. To verify that the indigenous Fe was involved in the improvement to the sludge dewaterability in Test Group I, Tests 4–7 were designed to test the effectiveness of H_2_O_2_ on sludge dewaterability when indigenous iron in the two sludges was diluted (described below). As part of Test Group II, ferrous salt was added to the two sludges at concentrations of 50 to 200 mg/L in Tests 8–12, to evaluate the impact of higher iron concentrations on sludge dewaterability improvement after treatment.

To reduce the indigenous Fe concentration in sludge, 200 mL of the sludge was mixed with 200 mL or 800 mL deionized water while the pH value was adjusted to 3.0 with sulfuric acid. The diluted sludge was then stirred by a magnetic stirrer (Heindolph Model MR 3000R) at 100 rpm for 30 min followed by gravity settling for 3 h. The corresponding amounts of supernatant (200 mL and 800 mL, respectively) of the diluted sludge were then removed. In this way, the concentration of indigenous Fe in both sludges was measured both before and after the dilution using method to be described in 2.3 After treatment, the iron concentration in SH decreased to 59% and 45% of the original concentration for the 200 mL and 800 mL dilutions, respectively, while the iron concentration in SL decreased to 71% and 66%, respectively.

The aim of Test Group III was to evaluate the effects of hydrogen peroxide concentration on the treatment efficiency. Based on the results from Test Groups I and II, an additional amount of iron, at iron concentrations of 100 and 200 mg/L for sludge SH and SL, respectively, was added to ensure that iron was not limiting. Various concentrations of hydrogen peroxide, ranging from 0 to 6000 mg/L were applied to identify the optimal hydrogen peroxide concentration.

For each batch test, 100 mL of WAS was transferred into a 250 mL glass flask. Ferrous salt was then added to adjust the iron concentration (as described above) and the pH of sludge was adjusted to 3 by adding sulfuric acid (25%). The flask was then mixed with a magnetic stirrer at 100 rpm for 30 min. 8 mL treated sludge was then sampled for CST measurement as described in 2.3. The sampling and CST measurements were repeated 5 times. The dissolved iron concentration both before and after each batch test was measured using the method described in 2.3.

### Chemical and physical analysis

The dewaterability of sludge was measured with a capillary suction timer (Trition-WPRL, Type 304). The sludge was filled in a 9 mL Stainless-steel funnel, and the time needed for permeating the filter paper by water from sludge (CST) was measured. CST was used as the indicator of sludge dewaterability.

To measure the dissolved iron concentration, the sludge was filtrated with 0.22 μm filters (millex), and the Fe concentration in the filtrate was analyzed with Inductive Coupled Plasma-Optical Emission Spectroscopy instrument (ICP-OES). For the measurement of the total Fe in sludge, the sludge sample was digested with 70% nitric acid for 15 min, and the dissolved iron was measured with ICP-OES.

The Total solids (TS) and Total volatile solids (TVS) concentrations in the original sludge were measured using APHA standard methods.

### Data analysis

Improvement of sludge dewaterability was assessed using the reduction percentage *R* (%) of CST. This is calculated as follows: 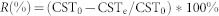
where *CST*_0_ was the CST of the original sludge, and *CST*_e_ was the CST after the treatment.

## Author Contributions

X.Z. designed part of the experiments and wrote the manuscript. G.J. and Q.W. designed part of the experiments. Z.Y. conceived the project and revised the manuscript.

## Figures and Tables

**Table 1:** The characteristics of Sludge I and Sludge II

	Sludge I (SH)	Sludge II (SL)
Iron (mg/L)	525	107
TS (g/L)	11.2 ± 0.2	12.3 ± 0.2
TVS (g/L)	8.5 ± 0.1	10.8 ± 0.3
CST (s)	91 ± 1.1	50 ± 0.7
pH	6.8	6.1

**Table 2:** Experimental design of the batch tests

Group	No.	Sludge	pH	H_2_O_2_ addition (mg/L)	Fe^2+^ addition (mg/L)
I. Preliminary tests	1	SH, SL	3	0	0
	2	SH, SL	NA^a^	3000	0
	3	SH, SL	3	3000	0
II. Effects of iron at various levels	4	200 SH/200 H_2_O^b^	3	3000	0
	5	200 SH/800 H_2_O^b^	3	3000	0
	6	200 SL/200 H_2_O^b^	3	3000	0
	7	200 SL/800 H_2_O^b^	3	3000	0
	8	SH, SL	3	3000	0
	9^c^	SH, SL	3	3000	50
	10	SH, SL	3	3000	100
	11	SH,SL	3	3000	150
	12^c^	SL	3	3000	200
III. Effects of hydrogen peroxide concentration	13	SH	3	0	100
	14	SH	3	170	100
	15	SH	3	340	100
	16	SH	3	760	100
	17	SH	3	1500	100
	18	SH	3	6000	100
	19	SL	3	0	200
	20	SL	3	40	200
	21	SL	3	80	200
	22	SL	3	130	200
	23	SL	3	170	200
	24	SL	3	340	200
	25	SL	3	760	200
	26	SL	3	1500	200
	27	SL	3	6000	200

^a^NA not adjusted. pH was approximately 6.8 (SH) and 6.1(SL).

^b^Indigenous iron was diluted in these tests through acid leaching, see text for details.

^c^Results from these two tests are also included in Group III tests to analyze effects of hydrogen peroxide concentration.

## References

[b1] Canales A., Pareilleux A., Rols J., Goma G. & Huyard A. Decreased sludge production strategy for domestic wastewater treatment. Water Sci Technol 30, 97–106 (1994).

[b2] Katsiris N. & Kouzeli-Katsiri A. Bound water content of biological sludges in relation to filtration and dewatering. Water Res 21, 1319–1327 (1987).

[b3] Hung W., Chang I., Lin W. & Lee D. Unidirectional freezing of waste-activated sludges: effects of freezing speed. Environ Sci Technol 30, 2391–2396 (1996).

[b4] Kepp U., Machenbach I., Weisz N. & Solheim O. Enhanced stabilisation of sewage sludge through thermalhydrolysis-three years of experience with full scale plant. Water Sci Technol 42, 89–96 (2000).

[b5] Wang F., Ji M. & Lu S. Influence of ultrasonic disintegration on the dewaterability of waste activated sludge. Environ Prog 25, 257–260 (2006).

[b6] Lee C. & Liu J. Enhanced sludge dewatering by dual polyelectrolytes conditioning. Water Res 34, 4430–4436 (2000).

[b7] Liao B., Allen D., Leppard G., Droppo I. & Liss S. Interparticle interactions affecting the stability of sludge flocs. J Colloid Interf Sci 249, 372–380 (2002). 10.1006/jcis.2002.8305 16290611

[b8] Erdincler A. & Vesilind P. Effect of sludge cell disruption on compactibility ofbiological sludges. Water Sci Technol 42, 119–126 (2000).

[b9] Tony M. A., Zhao Y., Fu J. & Tayeb A. M. Conditioning of aluminium-based water treatment sludge with Fenton's reagent: Effectiveness and optimising study to improve dewaterability. Chemosphere 72, 673–677 (2008). 18457862 10.1016/j.chemosphere.2008.03.032

[b10] Liu J. *et al.* Extracellular polymers of ozonized waste activated sludge. Water Sci Technol 44, 137–142 (2001). 11794644

[b11] Lu M.-C., Lin C.-J., Liao C.-H., Huang R.-Y. & Ting W.-P. Dewatering of activated sludge by Fenton's reagent. Adv Environ Res 7, 667–670 (2003). 11794674

[b12] Buyukkamaci N. Biological sludge conditioning by Fenton's reagent. Process Biochem 39, 1503–1506 (2004).

[b13] Neyens E., Baeyens J., Weemaes M. & De Heyder B. Advanced biosolids treatment using H2O2-oxidation. Environmen Eng Sci 19, 27–35 (2002).

[b14] Georgi A. *et al.* Humic acid modified Fenton reagent for enhancement of the working pH range. Appl Catal: Environ 72, 26–36 (2007).

[b15] Zhou X. *et al.* A review on sludge conditioning by sludge pre-treatment with a focus on advanced oxidation. RSC Adv 92, 50644–50652 (2014).

[b16] Kim T.-H. *et al.* Disintegration of excess activated sludge by hydrogen peroxide oxidation. Desalination 246, 275–284 (2009).

[b17] Ganigue R., Gutierrez O., Rootsey R. & Yuan Z. Chemical dosing for sulfide control in Australia: An industry survey. Water Res 45, 6564–6574 (2011). 22018528 10.1016/j.watres.2011.09.054

[b18] Fytianos K., Voudrias E. & Raikos N. Modelling of phosphorus removal from aqueous and wastewater samples using ferric iron. Environ Pollut 101, 123–130 (1998). 15093105 10.1016/s0269-7491(98)00007-4

[b19] Rasmussen H. & Nielsen P. H. Iron reduction in activated sludge measured with different extraction techniques. Water Res 30, 551–558 (1996).

[b20] González A. *et al.* Development of a non-conventional sorbent from fly ash and its potential use in acid wastewater neutralization and heavy metal removal. Chem Eng J 166, 896–905 (2011).

[b21] Karthik M. *et al.* Biodegradability enhancement of purified terephthalic acid wastewater by coagulation–flocculation process as pretreatment. J hazard mater 154, 721–730 (2008). 18054427 10.1016/j.jhazmat.2007.10.085

